# Professionals’ attitudes towards the use of cognitive enhancers in academic settings

**DOI:** 10.1371/journal.pone.0241968

**Published:** 2020-11-20

**Authors:** Sanyogita (Sanya) Ram, Bruce Russell, Carl Kirkpatrick, Kay Stewart, Shane Scahill, Marcus Henning, Louise Curley, Safeera Hussainy

**Affiliations:** 1 Centre for Medicine Use and Safety, Faculty of Pharmacy and Pharmaceutical Science, Monash University (Parkville Campus), Parkville, Australia; 2 School of Pharmacy, Faculty of Medical and Health Sciences, University of Auckland, Auckland, New Zealand; 3 Clinical Pharmacy, School of Pharmacy, University of Otago, Dunedin, New Zealand; 4 Centre for Medical and Health Sciences Education, Faculty of Medical and Health Sciences, University of Auckland, Auckland, New Zealand; 5 Department of General Practice, School of Primary and Allied Health Care, Faculty of Medicine, Nursing and Health Sciences Monash University, Notting Hill, Victoria, Australia; The University of Sydney School of Pharmacy, AUSTRALIA

## Abstract

**Introduction and aims:**

The non-medical use of prescription stimulants such as methylphenidate, dexamphetamine and modafinil is increasing in popularity within tertiary academic settings. There is a paucity of information on awareness, attitudes, and acceptability by professionals of use in this context. This study aimed to investigate professionals’ knowledge of and attitudes towards the use of cognitive enhancers (CEs) in academic settings, and their willingness to use a hypothetical CE.

**Design and methods:**

A mail survey was sent to doctors, pharmacists, nurses, accountants and lawyers in New Zealand. These disciplines were chosen as they require professional registration to practice. The questionnaire comprised four sections: (1) demographics, (2) knowledge of CEs, (3) attitudes towards the use of CEs, and (4) willingness to use hypothetical CEs.

**Results:**

The response rate was 34.5% (414/1200). Overall, participants strongly disagreed that it was fair to allow university students to use CEs for cognitive enhancement (Mdn = 1, IQR: 1,3), or that it is ethical for students without a prescription to use cognitive enhancers for any reason (Mdn = 1, IQR: 1,2). Professions differed in their attitudes towards whether it is ethical for students without a prescription to use CEs for any reason (*p* = 0.001, H 31.527).

**Discussion and conclusion:**

Divergent views and lack of clear consensus within professions and between professionals on the use of CEs have the potential to influence both professionals and students as future professionals. These divergent views may stem from differences in the core values of self-identity as well as extrinsic factors of acceptability within the profession in balancing the elements of opportunity, fairness and authenticity in cognitive enhancement. Further research is required to inform the development of policy and guidelines that are congruent with all professions.

## Introduction

The use of prescription stimulant medicines such as modafinil, amphetamines, and methylphenidate for cognitive enhancement is increasing, especially within tertiary academic environments [1, 2]. The lifetime prevalence of cognitive enhancer (CE) use amongst those students attending university has been reported to range from 1.2% to 34% [3–14]. This includes those students in all tertiary and college settings. CEs are used by healthy individuals in academic settings to try to improve concentration, increase alertness, stay awake longer or perform better academically. Methylphenidate is clinically indicated for Attention Deficit Hyperactivity Disorder (ADHD) and increases synaptic levels of dopamine and noradrenaline by blocking their respective reuptake transporters and consequently acting as a central nervous system stimulant. A systematic review conducted by Repantis *et al*. (2010) concluded that although studies showed a positive effect of methylphenidate on the memory of healthy individuals, further studies were warranted to determine its capacity [15]. While studies have shown cognitive- enhancing effects of modafinil in sleep-deprived individuals, it is unclear to what extent this extends to non-sleep- deprived individuals [15]. While there is continued debate on the whether CEs have an effect on healthy individuals, they continue to be used by healthy individuals for cognitive enhancement [3–14].

Despite there being numerous studies amongst tertiary students exploring their attitudes and motivations to use CEs, there is a paucity of information on awareness and acceptability of CE use in academic settings by professionals. It is recognised that use of CEs may extend beyond student life and into the workforce [16]. A survey of surgeons at five international conferences in 2011 reported that 8.9% of surgeons had used a prescription or illicit drug as a CE at least once in their lifetime [17]. In addition, an informal, online survey conducted by *Nature*, ‘Look who’s doping’, received responses from 1,400 respondents from 60 countries. While specific details of the respondents’ characteristics were not supplied, it was reported that one in five respondents had used drugs for non-medical reasons to help focus, enhance concentration or memory [18].

The decision to use CEs is influenced by personal attitudes towards the use of CEs as well as the wider attitudes of social networks. Similar to substance abuse where group norms around social acceptance dictate patterns of use [19], the use of CEs is enhanced by the influence of social networks and attitudes towards use. Social networks and friends are the main sources of information on CEs [20]. Family and friends are also important sources for obtaining CEs [6, 21, 22]. Maier *et al*. reported that users of CEs had a smaller social network and exhibited less prosocial behavior in social interaction tasks [23]. The influence of peers and the normalisation of CE use amongst social networks may provide the impetus to use CEs [24]. Judson *et al*. found that attitudes towards the ethics and perceived norms of CE use predicted use [25]. Lesser concerns about adverse health effects, ethics of use and perceived control, and higher perceived positive subjective norms were also related to illicit use among non-prescription holders [25]. A survey of students enrolled within professional courses namely medicine, pharmacy, nursing, accounting and law reported that those who perceived CE use to be socially and ethically acceptable were more likely to use CEs (OR 1.56, CI 1.153–2.105, *p* = 0.004) [20]. Beyond use amongst students, qualitative studies have suggested that the social context within which CEs are normalised, accessed and contextualised, which is important for framing the acceptability of CEs, especially in high pressured and competitive environments [26, 27].

Understanding public attitudes towards cognitive enhancement are fundamental to the development of acceptable, reasonable and effective policy [28]. A study exploring the attitudes of parents of university students and healthcare providers reported confusion and a lack of awareness of the prevalence of CE use by students [29]. Physicians’ views about prescribing and use of CEs pivot on the delineation between the treatment of a condition compared to enhancement, suggesting a trade-off between safety, harm and benefit [30–32]. Forlini and Racine (2009) reported that healthcare providers focussed their discussion of cognitive enhancement on health consequences and health risks [33]. However, there is a level of ambivalence towards what ought to be done about CE use [29]. Forlini *et al*. (2012) contend that professional policies may be sidestepping the values of stakeholders and call for a collaboration of professional associations with the humanities for a joint deliberation on the moral praiseworthiness of cognitive enhancement and pay closer attention to the divergence of fundamental values that has a broader impact on health and education [34].

The literature highlights the complexity of understanding the decision to use CEs; it is not a discrete and detached choice that occurs in isolation without consideration of the factors that influence the decision [20]. The biopsychosocial systems model of addiction proposes that intersecting biological, psychosocial, social and systemic properties are fundamental features of substance use [19]. It posits that knowledge of substance use occurs at the intersection of the subjective and the objective and not just as an independent reality [19]. This research focusses on the social and systems dimensions and seeks to explore the acceptability of CE use. Exploring how professionals, having undergone tertiary studies, view the use of CEs in the tertiary academic setting and whether they advocate the use of CEs, will help comprehend perspectives and endorsement of CE use.

### Aim

This research aims to investigate professionals’ awareness and perceived knowledge of the use of CEs in academic settings, and their attitudes towards and willingness to use a hypothetical CE.

## Methods

A mail survey was sent to a random sample of 200 professionals from each profession of general practitioners, psychiatrists, pharmacists, nurses, accountants and lawyers in New Zealand. General practitioners are medical practitioners who have specialised in the discipline of general practice, a clinical speciality orientated to primary care. These professions aligned with the academic disciplines of students we previously surveyed in a separate study [35]. These disciplines were chosen as they require registration to practice as a professional. The prevalence of CE use amongst students prompted the need to explore this phenomenon amongst practising professionals. The contact details of lawyers, accountants, nurses, general practitioners, psychiatrists and community pharmacists were obtained from either their respective registration authorities when permitted for research purposes or through publicly available online databases. Completion of the questionnaire and sending it back to the research team was deemed as consent to participate.

The questionnaire comprised four sections:

- demographics,
- knowledge of CEs,
- attitudes towards the use of CEs,
- willingness to use a hypothetical CE.

An additional section was included for potential prescribers of CE. Questions in Sections A and B were drawn from earlier research exploring student use of and attitudes towards CEs [6]. Section C was developed from a questionnaire used to explore attitudes towards CEs [20, 36], and employing questions that were deemed relevant to seek the attitudes of professionals. Each statement was followed by a seven-point Likert-type scale, ranging from strongly disagree (1) to strongly agree (7).

A draft version of the questionnaire was piloted with eight participants: four doctoral candidates, two psychiatrists, an emergency consultant, and a pharmacist. Feedback received included comments on the clarity of questions, the number of questions to complete and duplication of questions in the questionnaire. The draft questionnaire was amended based on the feedback provided. The study was approved by the University of Auckland Human Ethics Committee (2015:012571) and the Monash University Human Research Ethics Committee (2016:CF15/2541–2015001029).

An abridged version of the Dillman protocol for conducting surveys was used to distribute the questionnaire and follow up participants [37]. A paper-based survey method was chosen over online mechanisms due to legal and ethical constraints in obtaining email addresses of potential participants and gaining access to participants’ electronic addresses. Following the initial mail-out, two further mail-outs were sent to non-responders at three weeks and seven weeks. If potential participants did not wish to receive follow up reminders, they were invited to send their uncompleted questionnaires back in the self-addressed envelopes. The questionnaire was not coded; however, the envelope was coded to allow tracking of responses and follow up of non-responders.

Data analysis was undertaken using the Statistical Package for the Social Sciences (SPSS) V19 (IBM, Chicago, IL, USA) analytical software. Descriptive statistics were used to analyse participant characteristics. The median ranks in agreement and range were calculated for each attitudinal statement. Non-parametric results of the median (Mdn) and interquartile range (IQR) are reported. Kruskal-Wallis test was used to test for differences in attitudes towards the use of CEs in academic settings by the five groups of professionals for each attitudinal statement. Pairwise comparisons were used to explore the nature of any differences in attitudes found among the professionals [38].

## Results

The response rate was 34.5% (414/1200). Responses were received from 24% (48/200) of Accountants, 54.5% (109/200) of pharmacists, 28% (56/200) of general practitioners, 29.5% (59/200) of nurses, 30.5% (61/200) of lawyers, and 40.5% (81/200) of psychiatrists. Just over half of the respondents were female (n = 225, 54.35%), within the 50 to 59 years age bracket (n = 133, 32.12%) and in practice for longer than 30 years (n = 109, 26.3%) (Table 1).

**Table 1:** Familiarity with cognitive enhancers (n = 414).

Participant Characteristics	Number of Respondents % (n)	Heard of CEs % (n)	Very Knowledgeable % (n)	Knowledgeable % (n)	Somewhat knowledgeable % (n)	Not very Knowledgeable % (n)
**Profession**						
Accountant	11.6 (48)	2.4 (10)	0	(0)	1.0 (4)	9.9 (41)
Pharmacist	26.3 (109)	21.7 (90)	0.2 (1)	2.7 (11)	7.7 (32)	15.5 (64)
GP	13.5 (56)	10.1 (42)	0.7 (3)	1.4 (6)	3.9 (16)	7.2 (30)
Nurse	14.3 (59)	7 (29)	0.2 (1)	0.5 (2)	2.2 (9)	11.1 (46)
Lawyer	14.7 (61)	2.2 (9)	0	0.7 (3)	0.5 (2)	13.3 (55)
Psychiatrist	19.6 (81)	17.4 (72)	1.4 (6)	5.3 (22)	7.2 (30)	5.3 (22)
Total	414	60.9 (252)	2.7 (11)	10.6 (44)	22.5 (93)	62.5 (258)*
**Age**						
20–24 years	5.8 (24)	1.9 (8)	0	0.5 (2)	0.5 (2)	1.9 (8)
25–29 years	2.9 (12)	5.1 (21)	0	0.5 (2)	2.2 (9)	3.1 (13)
30–39 years	16.2 (67)	10.6 (44)	0.2 (1)	0.5 (2)	4.3 (18)	10.9 (45)
40–49 years	23.7 (98)	13.3 (55)	0.7 (3)	2.2 (9)	4.8 (20)	15.7 (65)
50–59 years	32.1 (133)	18.6 (77)	1.2 (5)	5.1 (21)	6.3 (26)	18.6 (77)
60–69 years	16.9 (70)	10.4 (43)	0.5 (2)	1.9 (8)	3.9 (16)	10.1 (42)
70 and above	2.4 (10)	1.0 (4)	0	0	0.5 (2)	1.9 (8)
**Gender**						
Female	54.3 (225)	33.3 (138)	1.0 (4)	3.6 (15)	11.6 (48)	37.0 (153)
Male	44 (182)	27.1 (112)	1.4 (6)	7.0 (29)	10.6 (44)	24.6 (102)
**Years of Practice**						
1–5 years	11.4 (47)	8.2 (34)	0.2 (1)	1.0 (4)	3.4 (14)	6.8 (28)
6–10 years	13 (54)	8.5 (35)	0.2 (1)	0.5 (2)	3.1 (13)	8.9 (37)
11–15 years	10.6 (44)	6.8 (28)	1.7 (7)	1.0 (4)	0.5 (2)	7.5 (31)
16–20 years	10.6 (44)	6.3 (26)	2.2 (9)	1.9 (8)	0	6.0 (25)
21–25 years	11.4 (47)	6.3 (26)	2.4 (10)	0.7 (3)	0.5 (2)	7.5 (31)
26–30 years	15.5 (64)	9.2 (38)	2.9 (12)	1.9 (8)	0.7 (3)	9.7 (40)
30 and above	26.3 (109)	15.2 (63)	6.5 (27)	3.4 (14)	0.5 (2)	15.2 (63)

Note: missing values for gender (n = 7, missing values for years of practice (n = 5).

*6 participants did not specify the prescription CEs that they had heard about but answered the question in relation to knowledge of CEs.

When participants were asked to list the CEs that they had heard about, the most commonly listed CEs were methylphenidate (n = 200, 48.3%), followed by dexamphetamine or methamphetamine (n = 131, 31.6%), modafinil (n = 83, 20%) and atomoxetine (n = 19, 4.6%).

Participants had first heard of CEs from scientific literature (23%) or the media (9.4%). Other sources included friends (4.3%), internet (3.1%), and family (1.4%). Most participants rated their knowledge of CE use in healthy individuals as either not very knowledgeable (n = 258, 62.3%), or somewhat knowledgeable (n = 93, 22.5%) (Table 1).

Overall, participants strongly disagreed that it was fair to allow university students to use CEs for cognitive enhancement (Mdn = 1, IQR: 1,3), to concentrate (Mdn = 1, IQR:1,2), to increase alertness/stay awake (Mdn = 1, IQR: 1,2) or to counteract the effects of other drugs (Mdn = 1, IQR: 1,2). Participants also strongly disagreed that it is ethical for students without a prescription to use cognitive enhancers for any reason (Mdn = 1, IQR: 1,2) and for students with a prescription to use CEs in excess or for purposes other than for which they were prescribed by a doctor (Mdn = 1, IQR: 1,2).

Participants agreed with the concern that taking medicines for cognitive enhancement, even as prescribed by a doctor, will adversely affect one’s health (Mdn = 5, IQR: 4,6). Participants indicated neutral scores on whether the use of CEs with a prescription (Mdn = 4, IQR: 2,4) or without a prescription (Mdn = 4, IQR: 3,4) is common at universities. They were also ambivalent as to whether CEs were effective for enhancement (Mdn = 4, IQR: 3,5).

There was a large variation in responses on whether it was safe for students with a prescription to use CEs as prescribed by a doctor for cognitive enhancement (Mdn = 4, IQR: 2,5) and on whether their colleagues believed that it is okay for students to use CEs as prescribed by a doctor for cognitive enhancement (Mdn = 3, IQR: 1,4).

Professions differed in their attitudes towards whether it is ethical for students without a prescription to use CEs for any reason (*p* = 0.001, H = 31.527) (Table 2). Pairwise comparison with adjusted *p-*values showed that pharmacists (Mdn = 1, IQR: 1,1) differed in their attitudes from nurses (Mdn = 1, IQR: 1,3) accountants (Mdn 1, IQR: 1,2), and lawyers (Mdn 2, IQR: 1,3). Professions also differed on whether it is fair to use CEs in excess of what is prescribed (p = 0.008, H = 15.749), whether it is ethical for students without a prescription to use CEs to concentrate (*p* = 0.001, H = 27.283), or to increase alertness/stay awake (*p* = 0.001, H = 41.622).

**Table 2:** Attitudes towards the use of CEs in academic settings.

								Pairwise Comparison	
	N	Median	Mode	Range	Interquartile Range	H	p–value		Adjusted Sig
a) It is fair to allow university students to use cognitive enhancers for cognitive enhancement.	408	1.00	1	6	1,3	17.054	0.004		
b) Students with lower academic performance should be allowed to use cognitive enhancers for cognitive enhancement.	408	1.00	1	6	1,3	5.601	0.347		
c) My colleagues believe that it is okay for students with a prescription to use cognitive enhancers as prescribed by a doctor for cognitive enhancement.	396	3.00	1	6	1,4	7.922	0.161		
d) It is ethical for students without a prescription to use cognitive enhancers for any reason.	407	1.00	1	6	1,2	31.527	0.001	Pharmacist (Mdn 1, Range 5, IQR: 1,1)–Nurse (Mdn 1, Range 6, IQR: 1,3)	0.006
								Pharmacist (Mdn 1, Range 5, IQR 1,1)–Accountant (Mdn 1, Range 5, IQR 1,2)	0.006
								Pharmacist (Mdn 1, Range 5, IQR 1,1)–Lawyer (Mdn 2, Range 5, IQR 1,3)	0.000
								General Practitioner (Mdn 1, Range 3, IQR 1,1)–Lawyer (Mdn 2, Range 5, IQR 1,3)	0.022
e) It is ethical for students with a prescription to use cognitive enhancers in excess or for purposes other than prescribed by a doctor.	408	1.00	1	6	1,2	15.749	0.008	Pharmacist (Mdn 1, Range 6, IQR 1,1)–Lawyer (Mdn 1, Range 5, IQR 1,3)	0.030
f) It is ethical for students without a prescription to use cognitive enhancers to concentrate.	407	1.00	1	6	1,2	27.283	0.001	Pharmacist (Mdn 1, Range 5, IQR 1,2)–Accountant (Mdn 2, Range 5, IQR 1,3)	0.029
								Pharmacist (Mdn 1, Range 5, IQR 1,2)–Lawyer (Mdn 2, Range 5, IQR 1,4)	0.000
								GP (Mdn 1, Range 4, IQR 1,2)–Lawyer (Mdn 2, Range 5, IQR 1,4)	0.005
								Psychiatrist (Mdn 1, Range 6, IQR 1,2)–Lawyer (Mdn 2, Range 5, IQR 1,4)	0.032
g) It is ethical for students without a prescription to use cognitive enhancers to increase alertness/stay awake.	408	1.00	1	6	1,2	41.622	0.001	Pharmacist (Mdn 1, Range 5, IQR 1,1)–Nurse (Mdn 1.5, Range 6, IQR 1,3)	0.017
								Pharmacist (Mdn 1, Range 5, IQR 1,1)–Accountant (Mdn 2, Range 5, IQR 1,3)	0.001
								Pharmacist (Mdn 1, Range 5, IQR 1,1)–Lawyer (Mdn 2, Range 5, IQR 1,3.5)	0.000
								GP (Mdn 1, Range 4, IQR 1,1)–Accountant (Mdn 2, Range 5, IQR 1,3)	0.031
								GP (Mdn 1, Range 4, IQR 1,1)–Lawyer (Mdn 2, Range 5, IQR 1,3.5)	0.001
								Psychiatrist (Mdn 1, Range 6, IQR 1,2)–Lawyer (Mdn 2, Range 5, IQR 1,3.5)	0.007
h) It is ethical for students with or without a prescription to use cognitive enhancers to counteract the effects of other drugs.	406	1.00	1	6	1,2	18.419	0.002	GP (Mdn 1, Range 4, IQR 1,2)–Lawyer (Mdn 2, Range 6, IQR 1,4)	0.002
								Psychiatrist (Mdn 1, Range 6, IQR 1,2)–Lawyer (Mdn 2, Range 6, IQR 1,2)	0.022
i) I believe it is safe for students with a prescription to use cognitive enhancers as prescribed by a doctor for cognitive enhancement.	403	4.00	4	6	2,5	5.584	0.349		
j) I believe the use of cognitive enhancers is effective for cognitive enhancement.	394	4.00	4	6	3,5	6.096	0.297		
k) I believe the use of cognitive enhancers is necessary for cognitive enhancement.	402	2.00	1	6	1,3	13.394	0.020		
l) I believe the use of cognitive enhancers by students with a prescription for cognitive enhancement is common at universities.	389	4.00	4	6	2,4	1.294	0.936		
m) I believe the use of cognitive enhancers by students without a prescription for cognitive enhancement is common at universities.	388	4.00	4	6	3,4	8.425	0.134		
n) I am concerned that taking cognitive enhancers for cognitive enhancement, even as prescribed by a doctor, will adversely affect one’s health.	404	5.00	7	6	4,6	9.474	0.092		

Each statement was followed by a seven-point Likert scale, ranging from strongly disagree (1) to strongly agree (7).

Significance values for Pairwise comparisons have been adjusted by the Bonferroni correction for multiple tests.

### Willingness to use a hypothetical CE

Participants were asked whether they would take a hypothetical prescription-only CE that shows proven efficacy, is approved by the regulatory authorities, and is devoid of significant side effects. As shown in Table 3, of the 398 participants who answered the question, 50% indicated that they would not use a hypothetical CE (n = 199), and only 11.6% (n = 46) said that they would. Hypothetically, the reasons they chose from a list where they could choose more than one option, included to improve concentration (n = 175), increase alertness (n = 123), alleviate pressure to perform better at work (n = 88), stay awake (n = 68), and for experimentation (n = 43). No significant differences were noted between whether participants had heard of a CE and whether they would be willing to take a hypothetical CE.

**Table 3:** Willingness to use a hypothetical CE.

	Yes			No			Maybe			Don’t wish to answer		
	%*	%**	n	%	%**	n	%	%**	n	%	%**	n
Accountant	0.5	4.2	(2)	5.1	43.8	(21)	4.8	41.7	(20)	0.2	2.1	(1)
Pharmacist	2.4	9.2	(10)	15.7	59.6	(65)	8.0	30.3	(33)	0.2	0.9	(1)
GP	2.2	16.1	(9)	6.8	50	(28)	4.1	30.4	(17)	0.2	1.8	(1)
Nurse	1.7	11.9	(7)	5.1	35.6	(21)	6.5	45.8	(27)	0.7	5.1	(3)
Lawyer	1.9	13.1	(8)	6.3	42.6	(26)	6.5	44.3	(27)	0.0	0	0
Psychiatrist	2.4	12.3	(10)	9.2	46.9	(38)	7.5	38.3	(31)	0.2	1.2	(1)
**Age**	0.0			0.0			0.0			0.0		
25–29 years	0.2		(1)	1.4		(6)	1.2		(5)	0.0		0
20–24 years	0.2		(1)	2.2		(9)	3.1		(13)	0.2		(1)
30–39 years	2.7		(11)	8.5		(35)	4.3		(18)	0.7		(3)
40–49 years	1.9		(8)	12.3		(51)	8.9		(37)	0.2		(1)
50–59 years	3.9		(16)	15.5		(64)	12.3		(51)	0.2		(1)
60–69 years	2.2		(9)	7.0		(29)	6.5		(27)	0.2		(1)
70 and above	0.0		0	1.2		(5)	1.0		(4)	0.0		0
**Gender**	0.0			0.0			0.0			0.0		
Female	5.3		(22)	24.4		(101)	22.2		(92)	1.2		(5)
Male	5.8		(24)	22.2		(92)	15.2		(63)	0.5		(2)
Missing	0.0			0.0			0.0			0.0		
**Years of Practice**	0.0			0.0			0.0			0.0		
1–5 years	1.0		(4)	5.1		(21)	5.1		(21)	0.2		(1)
6–10 years	1.4		(6)	6.8		(28)	4.6		(19)	0.2		(1)
11–15 years	1.4		(6)	4.8		(20)	4.1		(17)	0.2		(1)
16–20 years	1.2		(5)	6.8		(28)	2.7		(11)	0.0		0
21–25 years	1.0		(4)	6.8		(28)	3.1		(13)	0.2		(1)
26–30 years	1.7		(7)	5.8		(24)	7.7		(32)	0.0		0
30 and above	3.4		(14)	11.8		(49)	9.7		(40)	0.2		(1)

* % of overall sample

** % of profession.

## Discussion

This comparative study brings to light attitudes of professionals (pharmacists, doctors, nurses, lawyers, and accountants) towards the use of CEs in academic settings. Although participants strongly disagreed that it was fair to allow university students to use CEs for cognitive enhancement, participants were ambivalent on whether they believed that the use of CEs by students with or without a prescription for CE was common at universities. While participants disagreed that it is ethical for students without a prescription to use CEs for any reason, there was variation in responses across professions on whether it was safe for students with a prescription to use CEs as prescribed by a doctor. This may stem from divergent views on whether it is acceptable to use CEs if they are prescribed for cognitive enhancement and a level of acceptance of safety and risk if a doctor has prescribed them.

These findings are consistent with those reported by Banjo *et*. *al*. that healthcare providers posit concerns on fairness and social injustice that CE use presents. Our data, however, is inconsistent with findings by Forlini *et*.*al*. that parents and healthcare providers reported a level of ambivalence toward CE use [29]. There is agreement that it is unfair to use CEs; however, there is ambivalence on whether it is acceptable to use CEs as prescribed by a doctor and divergent views on whether it will adversely affect one’s health if used as prescribed. The acceptance of the use of CEs as prescribed may be seen as legitimising its use [39]. Students have expressed security in the safety of CEs as methylphenidate is a prescribed medication, not a street drug, and is safe because it has gone through extensive testing by pharmaceutical companies and is prescribed by medical professionals [24, 29]. Forlini *et*. *al*. contend that a multidirectional approach in interpreting ambivalence may actually reveal much deeper discomfort and moral unease with the wider social impact of CE use [29]. Peterson *et al*. categorised general practitioner attitudes towards the use of CEs as rejectors, navigators or acceptors, with more than half expressing comfort with optimising students capabilities [32]. Greater perceived knowledge has been related to a reduced perception of health risks with greater emphasis on alleged effectiveness and legality, rather than on risks for health [40]. This indicates divergent views and a lack of consensus on attitudes towards CEs.

There were differences amongst the professions on whether it was ethical to use CEs. Pharmacists more strongly disagreed than nurses, accountants, and lawyers that it is ethical for students without a prescription to use CEs for any reason. The social and psychosocial factors within professions may play a significant role in translating these attitudes towards CEs. For example pharmacists tend to affiliate more with the non-maleficent approach, with their primary goal being to reduce risk of harm and therefore they may consistently take a risk-averse approach [41]. Healthcare professionals differed from accountants and lawyers in their attitudes towards whether it was ethical to use CEs to increase alertness and wakefulness, with healthcare professionals having a more negative attitude. These differences may stem from personal concepts of self-identity and authenticity as well as challenges to the fundamental values held by the individual and the community [34]. These divergent views and lack of clear consensus within and between professions on the use of CEs have the potential to influence both the professionals and students as future professionals. This research provides insight into the attitudes towards the use of CEs in academic settings by professions and discourse necessary for the development of policy and guidelines that are congruent among professions.

When asked whether they would take a hypothetical prescription-only CE with proven efficacy, approved by regulatory authorities, and devoid of significant side effects, the hypothetical willingness to use CEs reported by professionals (11.6%) was higher than prevalence reported by students [35]; however, the reasons for use are similar amongst professionals and students. Most participants indicated that they would not take a hypothetical prescription CE even under these circumstances.

The hypothetical prevalence is higher than lifetime prevalence (6.6%) reported at a New Zealand tertiary institution but is within the range of 1.2% to 34% reported amongst college students [3–13]. Reasons for hypothetical use by professionals were consistent with students’ explanations for use, such as to improve concentration, increase alertness, stay awake longer or perform better academically. Professionals also indicated hypothetical use may occur in response to the pressure to perform at work.

## Limitations

As reported earlier, the data collected may be affected by underreporting, social desirability bias or unwillingness to disclose true attitudes [42]. The low response rate (34.5%) may hinder these results from being generalisable. The comparison between hypothetical prevalence and self-reported use is difficult, as it is unclear whether self-reports of hypothetical use would eventuate in actual use and self-reported data is based on memory and willingness to disclose. Hypothetical rather than actual use was explored in this study due to the sensitivity of the topic and concern for privacy, anonymity and confidentiality of reports and may not reflect actual behaviours and attitudes towards CE use due to social desirability of responses [43].

## Conclusion

This study brings to light the attitudes of professionals (pharmacists, doctors, nurses, lawyers, and accountants) towards the use of CEs in academic settings. While there is agreement that it is unfair for students to be allowed to use CEs for cognitive enhancement, there is ambivalence about the safety and use of CEs if prescribed by a doctor. Professionals differed in their attitudes towards CEs and this may stem from differences in the core values of self-identity as well as extrinsic factors of acceptability within the profession in balancing the elements of opportunity, fairness and authenticity in cognitive enhancement. Further research to explore the social and pyschosocial norms within professions, the reasons for ambivalence and the influence of the profession on endorsing or curbing the use of CEs is warranted.
